# Senescent cardiomyocytes contribute to cardiac dysfunction following myocardial infarction

**DOI:** 10.1038/s41514-023-00113-5

**Published:** 2023-06-14

**Authors:** Rachael E. Redgrave, Emily Dookun, Laura K. Booth, Maria Camacho Encina, Omowumi Folaranmi, Simon Tual-Chalot, Jason H. Gill, W. Andrew Owens, Ioakim Spyridopoulos, João F. Passos, Gavin D. Richardson

**Affiliations:** 1 Vascular Medicine and Biology Medicine Theme, Biosciences Institute, Newcastle University, Newcastle upon Tyne, UK; 2 Vascular Medicine and Biology Medicine Theme, Translational and Clinical Research Institute, Newcastle University, Newcastle upon Tyne, UK; 3 Department of Physiology and Biomedical Engineering, Mayo Clinic, Rochester, MN USA; 4 Robert and Arlene Kogod Center on Aging, Mayo Clinic, Rochester, MN 55905 USA

**Keywords:** Senescence, Cardiovascular diseases

## Abstract

Myocardial infarction is a leading cause of morbidity and mortality. While reperfusion is now standard therapy, pathological remodelling leading to heart failure remains a clinical problem. Cellular senescence has been shown to contribute to disease pathophysiology and treatment with the senolytic navitoclax attenuates inflammation, reduces adverse myocardial remodelling and results in improved functional recovery. However, it remains unclear which senescent cell populations contribute to these processes. To identify whether senescent cardiomyocytes contribute to disease pathophysiology post-myocardial infarction, we established a transgenic model in which p16 (CDKN2A) expression was specifically knocked-out in the cardiomyocyte population. Following myocardial infarction, mice lacking cardiomyocyte p16 expression demonstrated no difference in cardiomyocyte hypertrophy but exhibited improved cardiac function and significantly reduced scar size in comparison to control animals. This data demonstrates that senescent cardiomyocytes participate in pathological myocardial remodelling. Importantly, inhibition of cardiomyocyte senescence led to reduced senescence-associated inflammation and decreased senescence-associated markers within other myocardial lineages, consistent with the hypothesis that cardiomyocytes promote pathological remodelling by spreading senescence to other cell-types. Collectively this study presents the demonstration that senescent cardiomyocytes are major contributors to myocardial remodelling and dysfunction following a myocardial infarction. Therefore, to maximise the potential for clinical translation, it is important to further understand the mechanisms underlying cardiomyocyte senescence and how to optimise senolytic strategies to target this cell lineage.

Myocardial infarction (MI), is the leading cause of death and disability in developed countries^1^. Even, with reperfusion therapy, pathological myocardial remodelling can impact patient health by progressively impairing cardiac function, leading to heart failure^2^. Several independent studies have shown that MI causes senescence in numerous myocardial cell types, including cardiomyocytes (CMs), fibroblasts and endothelial cells^3,4^ (reviewed;^5,6^). Post-MI treatment with mechanistically diverse senolytic compounds, reduces senescent cell number, decreases inflammation, and improves heart function, suggesting that senescence contributes to disease pathophysiology^4,7^. Although the role of fibroblast senescence has been studied in this disease setting^8^ it remains unclear if senescent CMs actively participate in disease pathophysiology. Indeed, it has been proposed that as CMs lack a meaningful regenerative capacity^9,10^, their elimination could in fact be detrimental and senotherapies improve outcomes despite, rather than as a result of CMs apoptosis^6,7^. Moreover, while senolytic treatment improves outcome following MI, studies have failed to address the possibility that the observed benefits are due to non-myocardial senescent cell elimination or peripheral off-target effects^6,7^. For example, the molecular pathways influenced by senolytics are not uniquely expressed by senescent cells^11^ and non-resident myocardial cell populations which contribute to remodelling, including T-lymphocytes^12,13^ and platelets^14^, are influenced by senolytic treatment^15,16^. To continue the development of effective senotherapies, it’s imperative that the contribution of individual senescent cell populations to disease progression is understood.

The cyclin dependant kinase inhibitor p16 plays a key role in regulating CM senescence. p16 is increased in CMs with age and in response to myocardial infarction^4,17^. Furthermore, in aged *INK-ATTAC* mice, pharmacogenetic clearance of p16, reduces the percentage of CMs expressing alternative markers of senescence, reducing myocardial hypertrophy, and improving heart function^17^. Therefore, to investigate the specific role of CM senescence in disease pathophysiology following MI with reperfusion we employed a transgenic model that allows the CM specific inactivation of *CDKN2A* (which encodes p16)^4^. CM-p16^**KO**^ and CM-p16^**WT**^ mice were subjected to cardiac ischaemia reperfusion (IR) and assessed 5 weeks post-surgery (Fig. 1a, b; Supplementary experimental procedures). No animals died post-surgically in either group, and no differences in weight were observed between groups both pre- and post-IR (Supplementary Fig. 1). At 5 weeks the peri-infarct region of CM-p16^**KO**^ mice contained significantly fewer p16-expressing CMs compared to CM-p16^**WT**^ controls, indicating that p16 was effectively knocked out in CMs (Fig. 1c, d). CMs expressing p21 (a senescence marker) were also reduced in the peri-infarct region of CM-p16^**KO**^ mice compared to CM-p16^**WT**^ (Fig. 1e, f). Additionally, cytokine array analysis identified a decrease in several proteins, including a significant reduction in interleukin-6 (IL-6) (*p* < 0.05), a decrease in interferon-gamma (INF-γ *p* = 0.08) and granulocyte colony-stimulating factor (G-CSF, *p* = 0.07) (Fig. 1g, h). Further, macrophage inflammatory protein 3 alpha (MIP-3βa) and IL-5 were reduced to undetectable levels in CM-p16^ko^ mice (Supplementary Table 1). Our results suggest that the infarcted myocardium of p16-CM^KO^ mice has decreased expression of several proteins associated with the senescence-associated secretory phenotype (SASP) and proteins with described roles in myocardial remodelling^18^. Although this attenuation in SASP was not as robust as when senescence was eliminated using a senolytic non-cell specific approach^4^, these findings may be expected due to the cell-specific nature of the clearance used in the current study, and the observed continued expression of p21 in a proportion of CMs. All cytokine array data are included in Supplementary Table 1.

**Fig. 1:**
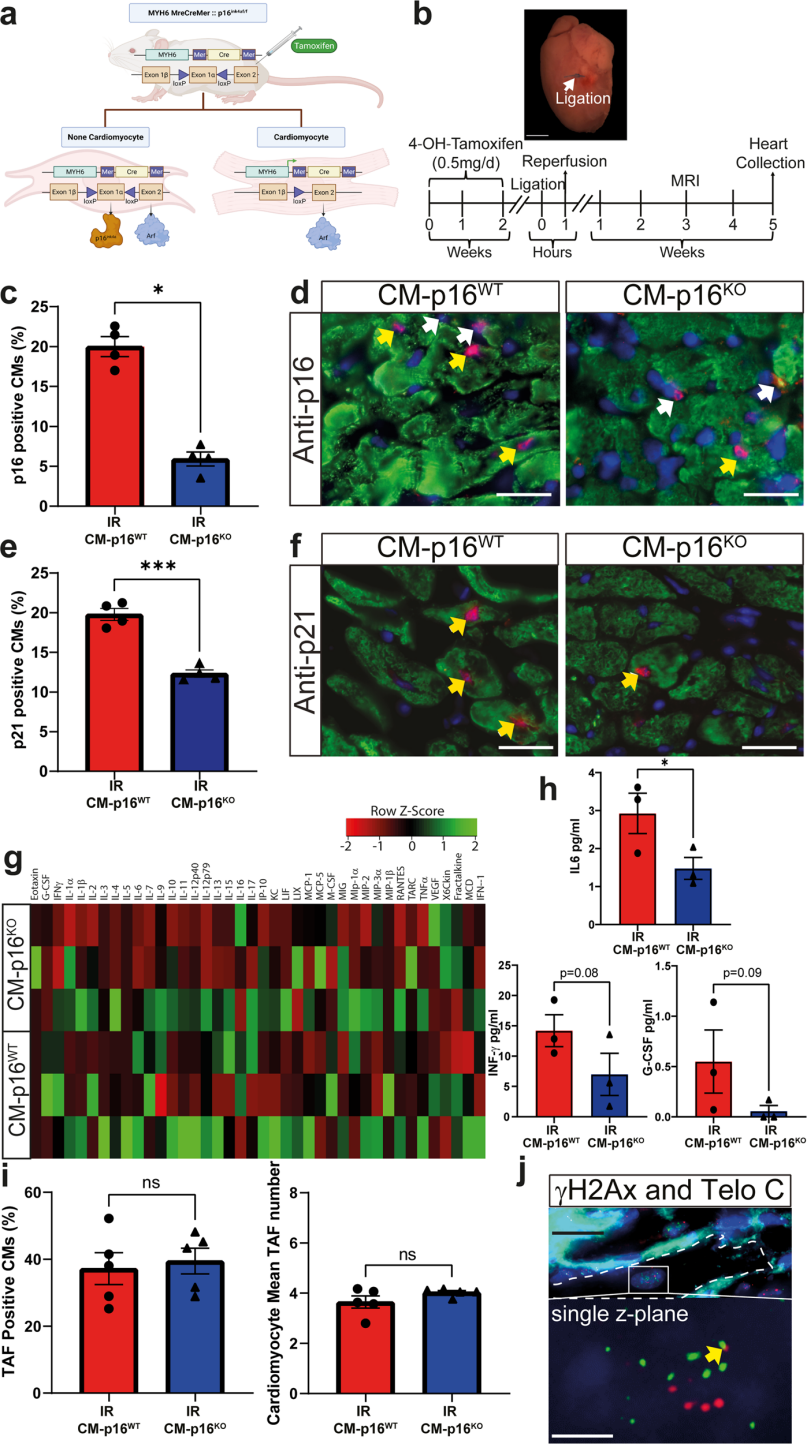
Cardiomyocyte specific knock out of p16 reduces cardiomyocyte senescence and senescence associated secretory phenotype. **a** Schematic of transgenic cardiomyocyte p16 knock-out mouse. **b** Experimental design. **c** Percentage p16^**+**^ CMs in the peri-infarct region at 5 weeks post IR. **d** Representative images of p16 staining. Yellow arrow, p16^**+**^ CM and white arrow p16^**+**^ interstitial cell (p16 red, Troponin-C green, DAPI blue), *n* = 4/group. Scale bars 20 µm. **e** Percentage p21^**+**^ CMs in the peri-infarct region at 5 weeks post IR. **f** Representative images of p21 staining. Yellow arrows, p21^**+**^ CM (p21 red, Troponin-C green, DAPI blue), *n* = 4 per group. Scale bars 20 µm. **g** Clustered heatmap of cytokine protein levels in LV myocardium. **h** Expression of individual protein levels in the LV myocardium of heart in the indicated experimental groups. **i** Total number of cardiomyocytes with TAF and mean of TAF per cardiomyocyte at 5 weeks post IR. *n* = 3/group. **j** Representative images of TAF, γH2AX co-localised with Telomere immuno-FISH (telo-FISH red, γH2AX green, WGA white). Images are obtained from the z-stacks of 10 μm sections. Yellow arrow indicates a TAF. Scale bars top panel 20 µm and bottom panel 2.5 µm. Data are mean ± SEM, *P < 0.05 ***P < 0.05 using Student’s *T* test or Mann–Whitney test.

Within CMs telomere foci of DNA damage (TAF) can trigger activation of p16 and p21 associated senescence pathways^17^. Our previous study demonstrated in the hearts of 3 month old healthy mice approximately 20% CMs contain at least 1 TAF^17^. Subsequent to IR 40% of CMs were observed to contain ≥1 TAF in both CM-p16^**WT**^ and CM-p16^**KO**^ mice, indicating IR induces DNA damage in the form of TAF, (Fig. 1i, j). However, as would be expected given that TAF induction is upstream of p16 activation there was no difference in mean TAF number or the percentage of CMs with TAF between the two groups (Fig. 1i). This is also suggestive that the degree of insult and stress as a result of IR was similar between the two groups.

While p21 expression was reduced in the CM-p16^KO^ group, this was not to the same extent as p16 expression, as despite an approx. 5-fold reduction in p16^+^ CMs only an approx. 2-fold reduction CM expressing p21 was observed (Fig. 1e, f). This data together suggests that, although p21 dependent senescence may still occur in CMs following inactivation of p16, CM senescence was indeed dampened. Several studies have demonstrated that p16 directly controls p53/p21 pathway activation. In fibroblasts, p16 specific siRNA abrogates p21 activation in response to DNA damage induction via UV light^19^, p16 stabilizes p21 mRNA through negative regulation of the mRNA decay-promoting AUF1 protein in several cell types^20^, and p16 has been demonstrated to regulate p53 expression at the protein level^21^. Together, these studies provide a mechanism that explains our observations that p16 inactivation suppresses p21 expression. It is also possible that p16 knockout reduces activation of the p53/p21 pathway via the reduction of oxidative stress thereby protecting against DNA damage. However, quantification with Dihydroethidium (DHE), identified no difference in superoxide levels in the peri-infarct region of hearts of CM-p16WT and CM-p16KO mice (Supplementary Fig. 2). This data together with our observations that DNA damage in the form of TAF was comparable between experimental groups, argues against reduced oxidative stress and DNA damage as a mechanism of p21 expression reduction.

To determine if senescent CMs contribute to maladaptive myocardial remodelling, and therefore impaired cardiac function after IR, mice were analysed using cardiac magnetic resonance imaging (MRI) (Fig. 2a, b). At 5 weeks post-IR CM-p16^**KO**^ mice had a significantly higher ejection fraction compared to CM-16^**WT**^ littermates (Fig. 2b). This was attributed to improved preservation of left ventricular (LV) systolic volume, as no significant difference in LV end diastolic volume was observed between the groups. A trend in improved stroke volume was also evident in CM-p16^**KO**^ mice (Supplementary Fig. 3). Senescence has been previously linked to pathological CM hypertrophy^17,22^. We therefore aimed to identify if improved maintenance of cardiac function was a result of the attenuation of pathological hypertrophy in the CM-16^KO^ hearts. No differences were observed in hypertrophy measured at an organ or cellular level as LV mass indexed to tibia length and mean CM area were consistent between experimental groups (Fig. 2b–d). This data suggests that, in the acute setting, senescence is not a leading driver of hypertrophy and alternative pathways, for example the angiotensin II and the renin-angiotensin-aldosterone system pathways may be more important^23^. Interestingly, scar size was significantly reduced in CM-p16^**KO**^ mice, suggesting that CM senescence contributes to this aspect of pathological remodelling after IR (Fig. 2e, f). This data together with the observed reduction in SASP expression in the myocardium of p16-CM^**KO**^ mice led us to hypothesise that CM senescence and SASP has a paracrine influence on surrounding non-CM cell populations. Supporting this hypothesis, CM-p16^**KO**^ mice demonstrated a significant reduction in p16 expressing and senescence associated-β-galactosidase positive interstitial cells in the myofibroblast rich peri-infarct at 5 weeks post IR (Fig. 2g–i). Furthermore, studies have demonstrated that senescence induction promotes myofibroblast differentiation and enhances collagen deposition^8,24^. This, together with our current study provides a mechanism by which senescent CMs promote scar formation and cardiac dysfunction following IR. However, it should be highlighted that senescence in the fibroblast population is not always detrimental. Fibroblast senescence is essential for wound-healing in skin^25^. Furthermore, systemic attenuation of senescence via knockout of p53 or p53 and p16 can result in exaggerated fibrosis in the heart following myocardial insult^8,24,26^. In these studies, increased fibrosis was attributed to continued proliferation, as in the absence of senescence, the fibroblasts do not exit the cell cycle. This data suggests that fibroblast senescence may be beneficial at a specific timing or condition after myocardial infarction.

**Fig. 2:**
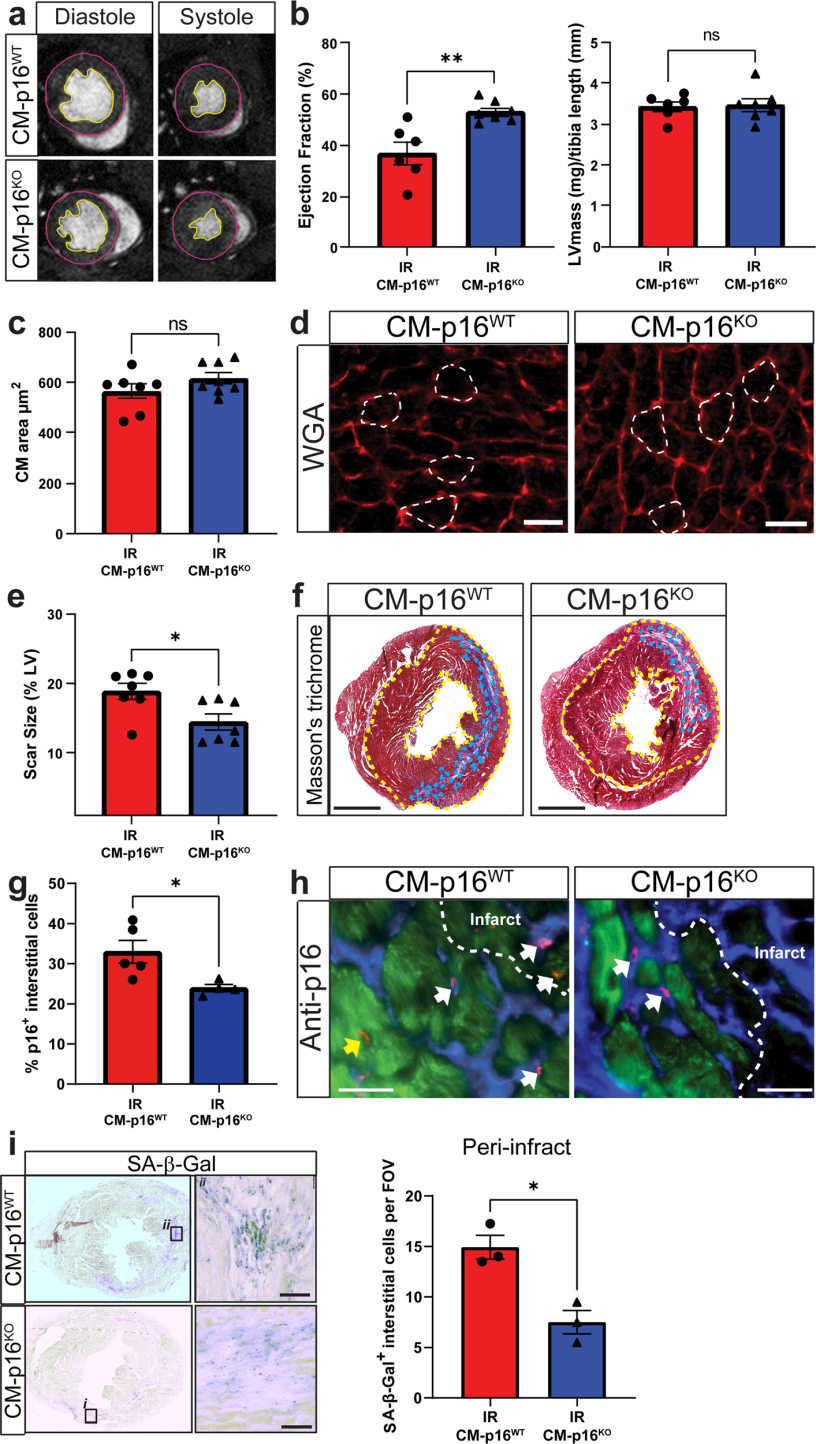
Cardiomyocyte specific inhibition of senescence improves functional outcome, reduces scar size and attenuates senescence in interstitial cells following IR. **a** Examples of individual short axis cine-MR images. **b** Ejection fraction and LV mass at 5 weeks post-IR, *n* > 6/group. **c** Mean CM cross-sectional area μm^2^. *N* = 7/group. **d** Representative images of WGA staining for quantification of CM area. Scale bars 20 µm. **e** Quantification of infarct size relative to total LV area. *N* = 7/group. Scale bars 1 mm. **f** Representative images of Masson’s trichrome staining. **g** Percentage p16^+^ interstitial cells in the peri-infarct region at 5 weeks post IR. **h** Representative images of p16 staining. Yellow arrows, p16^+^ CMs and white arrows, p16^+^ interstitial cells (p16 red, Troponin-C green, DAPI blue), *n* = 4/group. Scale bars 20 µm. **i** Representative image of SA-β-gal staining at 5 weeks post IR and quantification of SA-β-Gal^+^ interstitial cells per field of view (FOV) in the peri-infarct region at 5 weeks post IR, *n* = 3. Scale bars 50 µm. Data are mean ± SEM. *P < 0.05, **P < 0.01 using Student’s *T* test or Mann–Whitney Test.

Collectively, this study demonstrates for the first time, that following IR, CM senescence is detrimental to cardiac function and promotes myocardial remodelling, adding to the growing body of evidence which suggests that post-mitotic cell senescence plays a crucial role in the pathophysiology of various diseases. The data also indicates that this is a result of SASP expression, which induces paracrine senescence and promotes fibrosis and scar production. This suggests a more in-depth study of CM SASP could uncover unique biomarkers that are more accurate in predicting myocardial dysfunction compared to SASP from other cell types found across multiple organ systems. In conclusion, it is crucial that future therapeutic approaches consider the significant contribution of senescent CMs to myocardial remodelling and dysfunction.

## Methods

### Ethics approval statement

All animal studies were conducted in accordance with the Guidance on the Operation of the Animals (Scientific Procedures) Act, 1986 (UK Home Office), and approved by the local ethics committee (Animal Welfare & Ethical Review Body) at Newcastle University.

### Transgenic mouse breeding and genotyping

Mice were purchased from Charles River (Charles River Laboratories International, UK) or the MRC Harwell Institute. To establish a mouse line that allows the cardiomyocyte-specific knock out of p16Ink4a, transgenic p16^**Ink4a**^ floxed mice ((Cdkn2atm2.1Nesh line (p16^**ink4af/f**^)^27^ were crossed with an inducible Cre line under activation of a cardiomyocyte-specific promoter (Myh6‐cre/Esr1 (αMHC-MerCreMer)). The αMHC-MerCreMer mice carry a fusion transgene of Cre recombinase flanked by Mer, a mutated oestrogen receptor ligand-binding domain, driven by the cardiac α‐myosin heavy chain promoter (encoded by Myh6). The p16^**ink4af/f**^ line carries loxP elements ∼3.5 kb upstream of 5′ to exon 1α and immediately downstream of 3′ to the p16^**Ink4a**^ exon 1α. To produce experimental and control animals for the studies, MerCreMer:^**+/−**^: p16+/f males were bred with MerCreMer:^**−/−**^: p16^**+/f**^ females to create αMHC-MercreMer:^**+/−**^: p16f/f (CM-p16^**KO**^) and littermates controls MerCreMer:^**−/−**^: p16^**f/f**^ (CM-p16^**WT**^). Mice were treated with 4-OH-tamoxifen (Sigma) dissolved in peanut oil at a concentration of 0.5 mg/day (I.P) for 2 weeks and subjected to cardiac ischaemia reperfusion. Using primers detailed in Supplementary Fig. 4, genotype, and excision of the p16Ink4a exon 1α was verified by PCR (Supplementary Fig. 5). Mice were culled by humane methods and the hearts were collected directly into 50 mM KCl, to arrest in diastole for downstream analysis.

### Cardiac ischaemia reperfusion

Male mice at 3–4 months of age were used in all studies. Intra-operative analgesia was induced by pre-treating mice with fentanyl/fluanison (0.4 ml/kg, Hypnorm), prior to anaesthesia using isoflurane, which was maintained using mechanical ventilation following endotracheal intubation (2.5% isoflurane/97.5% oxygen, 130–140 stroke rate, stroke volume initially 5 ml/kg – increased to 7.5 ml/kg post-thoracotomy). At the fourth-intercostal space, left-side thoracotomy was executed to allow partial removal the pericardium and enable a 7–0 prolene suture to be placed around the LAD and loosely tied. An infarction was induced by inserting 2 mm PE-10 tubing into the suture loop and tightening the suture knot to terminate blood flow for 60 min. The tubing was removed, the chest cavity closed and 0.05 mg/kg, Vetergesic was provided as analgesia. During LAD-ligation surgery, it is possible that total occlusion is not accomplished resulting in a small infarct that does not model MI. Conversely, it is possible that reperfusion following 60 min ligation is not successful resulting in a permanent ligation. To control for this, prior to any other analysis, scar size was measured (as described below) in all animals in a blinded fashion and animals with a scar size of >25% or <10% were excluded from all analysis. All hearts removed from the study are shown in Supplementary Fig. 6. These exclusion criteria are based on the published data of scar size when using the permanent or ischaemia reperfusion models from 16 studies as reviewed^28^.

### Microscopy and image analysis

All images were acquired using Axio Imager (Zeiss) and analysed using ZEN 2.3 (Zeiss). The peri-infarct region was defined as the region proximal to the infarct in the left ventricle. For quantification studies a minimum of 20 images/mouse were analysed >5 sections in comparable regions.

### Scar size quantification

Masson’s trichrome staining was performed in order to visualise the infarct.29 Hearts were sectioned into 5 sets of slides, 10 slides per set. Sections were fixed for 1 h in 4% PFA followed by overnight incubation in Bouin’s solution (HT10132, Sigma), both at room temperature. Slides were washed in tap water, and nuclei were labelled with Weigert’s Haematoxylin solution (HT1079, Sigma). Cytoplasm staining was achieved by staining with Beibrich Scarlet-Acid Fuchsin Solution (HT151, Sigma) followed by an incubation in phosphotungstic/phosphomolybdic acid solution with ddH2O in a 1:1:2 solution respectively. Slides were incubated in Aniline Blue Solution (b8563, Sigma), washed in ddH2O and placed in 1% glacial acetic acid. Sections were dehydrated via an EtOH gradient washed in Histoclear (HS-202, National Diagnostics) and mounted using Histomount (HS-103, National Diagnostics). Each set of slides from each individual heart were imaged and analysed using the Leica Digital Image Hub. The left ventricular area was calculated by measuring the epicardial area and subtracting the endocardial area. The infarct area was then measured and the percentage of left ventricle that was infarcted was calculated as a percentage of the total left ventricle.

### Histology and immunohistochemistry

Hearts were either cryo-embedded or embed in paraffin wax. For cryo-embedding, hearts were incubated in 30% sucrose at 4 °C with agitation for 6 h. Tissues were then embedded in optimal cutting temperature compound (OCT) (Agar Scientific Ltd, UK), and stored at −80 °C. For wax embedding, hearts were first fixed in 4% paraformaldehyde (PFA) at 4 °C overnight with gentle agitation. Tissues were then dehydrated and embedded in paraffin. The hearts were sectioned to produce 10 μm thick transverse sections on a cryostat or 5 μm thick transverse sections on a microtome and collected as sister sections on positively charged Histobond slides (Marienfeld). Paraffin-embedded heart sections underwent de-paraffination and antigen-retrieval (0.01 M Citrate buffer (pH6.3)) prior to staining. For immunofluorescence, sections were fixed in 4% PFA for 20 min and permeabilised in a 0.5% Triton-X solution and blocking was via 10% Foetal Calf Serum. Slides were incubated with primary antibody overnight at 4 °C or for 1 h at room temperature. After incubation with the appropriate secondary antibody slides were mounted in Vectashield Antifade Mounting Medium containing DAPI (H-1500, VectorLab). Primary antibodies used: rat anti-p21 (Hugo291, Abcam), goat-anti-Troponin C (ab30807, Abcam), rabbit anti-p16Ink4a (100401170, Rockland). Secondary antibodies used were donkey anti-rat (1:200, 594 nm, A21209, Life Technologies), donkey anti-rabbit (1:500, 594 nm, R37119, Life Technologies) and donkey anti-goat (1:500, 488 nm, A11055, Life Technologies); containing DAPI (1:500, MBD0015, Sigma) for 45 min at room temperature. All quantifications were performed blinded to treatment and genotype using digital image analysis (ImageJ; U.S. National Institutes of Health; http://rsbweb.nih.gov/ij/). Quantification of p16 and p21 was restricted to the peri-infarct region. A minimum of 20 images/heart over 5 individual sections were taken within this peri-infarct area (Total approx. 6000 cells analysed per heart. For each image, the total number of CMs (identified by Trop-C expression) and the number of CMs expressing the protein of interest was quantified, allowing the percentage CM expressing each protein to be calculated. Similarly, the number of interstitial cells (identified as DAPI+ Trop-C-) expressing the protein of interest was quantified allowing the percentage expression to be calculated. To quantify cardiomyocyte hypertrophy, sections were stained with wheat germ agglutinin (WGA) labelling (Alexa Fluor® 647 conjugate, W32466, Invitrogen). CM hypertrophy was assessed by cross‐sectional area of cell membranes labelled with WGA, and troponin C was used to identify cardiomyocytes^29^. Only CMs in the peri-infarct region of the left ventricle free wall were analysed. To control for tissue orientation, only cardiomyocytes that were surrounded by capillaries all displaying a cross‐sectional orientation were analysed. To detect ROS, unfixed frozen tissue sections were incubated with 10 µM dihydroethidium (DHE, Sigma) for 30 min at room temperature, after which the peri-infarct zone was imaged. Generation of superoxide radicals was demonstrated by a red fluorescent signal and fluorescence intensity was quantified on acquired digital images using Image J software.

### Immuno‐FISH

Telomere-associated DNA damage foci (TAF) were detected by performing Immuno-FISH, as described^30^, on cryo-embedded heart sections. Briefly, sections that were labelled with rabbit monoclonal anti-γH2Ax (20E3, Cell Signalling Technology, 9718) and following secondary labelling with goat anti-rabbit IgG biotinylated (VectorLab, PK-6101), sections were fixed with methanol: acetic acid (3:1), dehydrated and incubated with PNA hybridisation mix with 5% blocking reagent (Roche) containing 2.5 μg/ml Cy3‐labelled telomere‐specific (CCCTAA) peptide nucleic acid probe (Panagene).

### Cytokine/Chemokine array

Portions of heart (superior to suture, affected RV and affected LV) were placed in 1 ml of RIPA buffer (R0278, Sigma) supplemented with protease inhibitors (58927091001, cOmplete ULTRA Tablets). Tissue was then homogenised. Protein content was normalised using a bicinchoninic acid assay, according to manufacturer’s instructions. Cytokine array was performed by Eve technologies using the Mouse Cytokine Array/Chemokine Array 44-Plex (MD44). Heat maps were generated with www.heatmapper.ca/expression and results displayed with Average Linkage Clustering method with Pearson Distance Measurement method.

### Senescence-associated β-galactosidase staining

Cryo-embedded sections were stained using the senescence associated β-galactosidase (SA-β-Gal) staining kit (Cell Signalling Technology, 9860) as per the manufacturer’s instructions with the following modifications for tissue sections. Slides were thawed at room temperature, fixed using the provided fixative solution for 15 min and washed three times in PBS. The β-galactosidase staining solution was prepared at pH 6 and added to each slide. Slides were incubated at 37 °C for 24 h. Once developed slides were washed with PBS, dehydrated in 95 and 100% ethanol solutions, washed in Histoclear and mounted with Histomount. All imaging was performed using an Axio Imager (Zeiss) and analysed using ZEN 2.3 (Zeiss). All images were taken using the same exposure time and were exported following an automated setting of the min/max values for brightness and contrast using Zen software.

### Magnetic resonance imaging and analysis

Magnetic Resonance Images (MRI) were generated at 5 weeks post-LAD ligation on the horizontal bore 7.0T Varian microimaging system (Varian Inc., Palo Alto, CA, USA), situated at the Campus for Ageing and Vitality, Newcastle University. Mice were anaesthetised using isoflurane (3.5% initially and once unconscious isoflurane reduced to 1.5% with 1 L/min oxygen) administered by facemask then placed on a specially designed stage with integrated electrocardiographic, cutaneous, temperature and respiratory monitors. To ensure that the orientation of the heart was optimal for imaging, a scout image was performed. Once correctly orientated, the whole heart was imaged using FLASH cine MR sequence. Each MR sequence is initiated by the R wave of the electrocardiogram (ECG), which corresponds to end diastole. Short, continuous axis slices with a 1 mm thickness were acquired so that the whole LV could be visualised. Images were analysed using Image J^31–34^. All images for each individual axis slice were combined to give a stack image showing the heart at that axis throughout the cardiac cycle. At the point of end diastole and end systole, the area of the epicardium and endocardium were measured for calculations of cardiac function.

### Statistical analysis

All analysis was performed in a blinded manner. All analysis was performed using GraphPad Prism 8.0. All data was first tested for normality to identify the appropriate statistical analysis. To test the hypothesis that p16 knock-out would reduce SASP expression we used a two-sample, 1-tailed T-test. All other data was tested using a 2-tailed T-test, or Mann–Whitney test as appropriate. *P* < 0.05 was considered as significant.

### Reporting summary

Further information on research design is available in the Nature Research Reporting Summary linked to this article.

## Supplementary information

Supplementary Figures

Reporting Summary

## Data Availability

All data generated or analysed during this study are included in this published article and its supplementary information files.
