# Author Correction: Senescent cardiomyocytes contribute to cardiac dysfunction following myocardial infarction

**DOI:** 10.1038/s41514-023-00115-3

**Published:** 2023-06-23

**Authors:** Rachael E. Redgrave, Emily Dookun, Laura K. Booth, Maria Camacho Encina, Omowumi Folaranmi, Simon Tual-Chalot, Jason H. Gill, W. Andrew Owens, Ioakim Spyridopoulos, João F. Passos, Gavin D. Richardson

**Affiliations:** 1 Vascular Medicine and Biology Medicine Theme, Biosciences Institute, Newcastle University, Newcastle upon Tyne, UK; 2 Vascular Medicine and Biology Medicine Theme, Translational and Clinical Research Institute, Newcastle University, Newcastle upon Tyne, UK; 3 Department of Physiology and Biomedical Engineering, Mayo Clinic, Rochester, MN USA; 4 Robert and Arlene Kogod Center on Aging, Mayo Clinic, Rochester, MN 55905 USA

**Keywords:** Senescence, Cardiovascular diseases

Correction to: *npj Aging* 10.1038/s41514-023-00113-5, published online 14 June 2023

In this article the text stated *P* > 0.05 should be *P* < 0.05. The original article has been corrected.

