# Supplementary material for: Senescent cardiomyocytes contribute to cardiac dysfunction following myocardial infarction
Source: NPJ Aging. 2023 Jun 14;9(1):15. doi: 10.1038/s41514-023-00113-5 (PMC10267185; doi:10.1038/s41514-023-00113-5)
Supplement: Supplementary file 1 — Supplementary Figures [file 41514_2023_113_MOESM1_ESM.pdf]

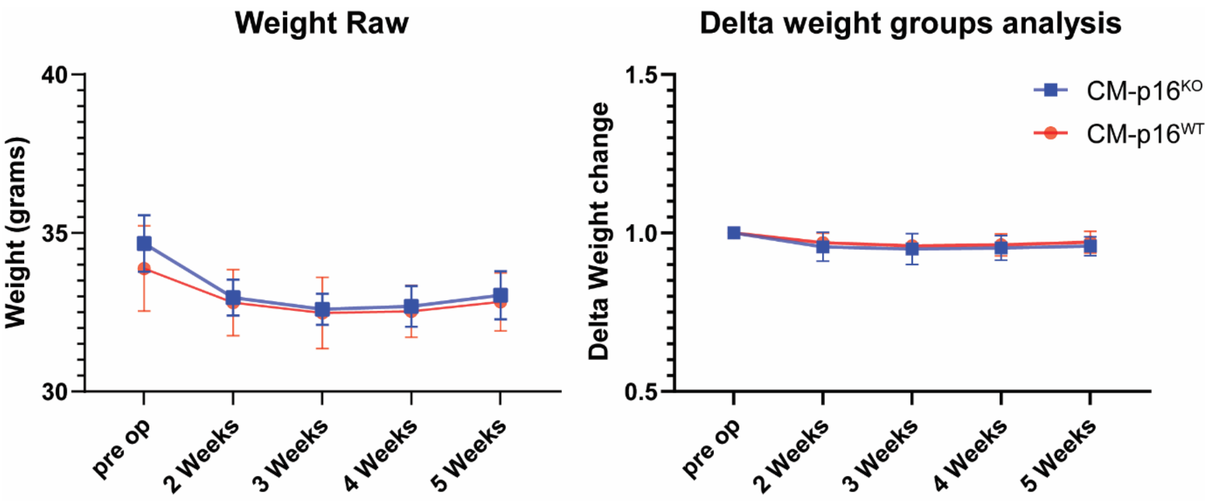

Supplementary Figure 1. **Body weight and delta change in body weight for all animals for the duration of the study.** No significant difference was observed in weights pre or post LAD-ligation between groups. No difference in weight change was observed between groups. Analysis performed by multiple student T-Test.

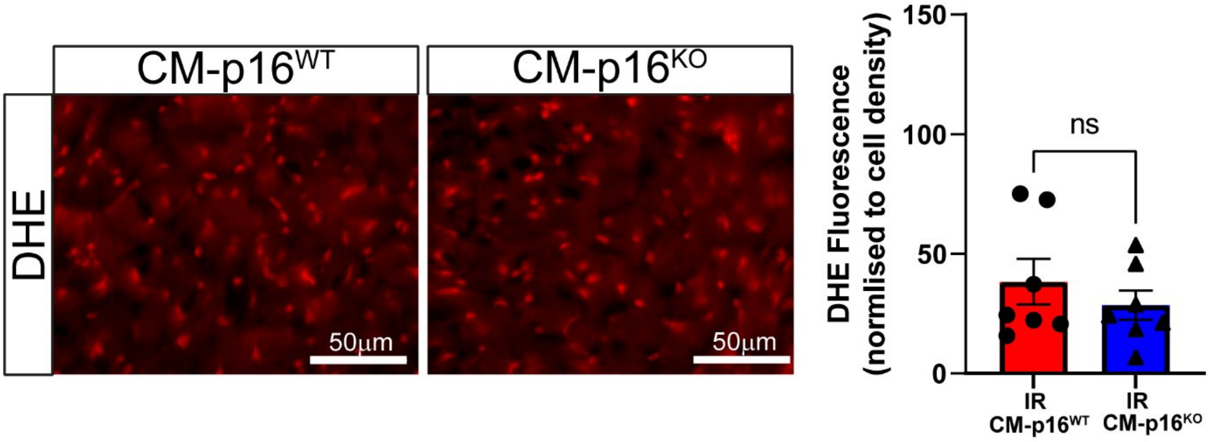

Supplementary Figure 2. **Superoxide levels post ischemia reperfusion.** Representative image of Dihydroethidium (DHE) staining at 5-weeks post LAD-Ligation. Quantification of DHE fluorescence intensity in the peri-infarct myocardium of CM-p16KO and CM-p16WT mice. n=7/group. All scale bars as indicated. Data are mean±SEM, analysis by Student T-Test.

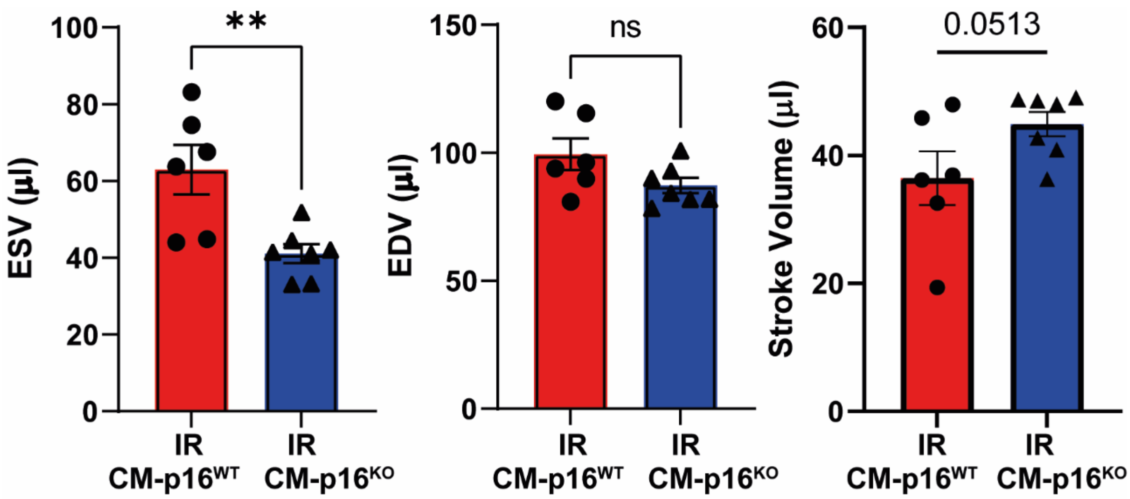

Supplementary Figure 3. **Magnetic resonance image analysis.** End diastolic volume (ESV), End systolic volumes (EDV) and stroke volume 5 weeks post-MI, n>6/group. Data are mean±SEM, **P<0.01. Using Mann-Whitney or student T-Test as appropriate.

Sequences for primers used for genotyping polymerase chain reactions.

**Name**	**Primer Sequence**
p16^{Ink4a} f/f F	GTATGCTATACGAAGTTATTAGGTACTGC
p16^{Ink4a} wild-type F	GTTTTGGAGCAGCAGGGATT
p16^{Ink4a} common R	CTATGTCAGATTTGGCTAGGGAGT
S1X-A Cre F	TAACCAGTGAAACAGCATTGCTG
S1X-B Cre R	GGACATGTTCAGGGATCGCCAGGCG
p16^{Ink4a} LCred F	TACCACAGTTTGAACAGCGTGA
p16^{Ink4a} LCred R	AACCAACTTCCTCCTTCCCC

Supplementary Figure 4. **Primer sequences used in the study.**

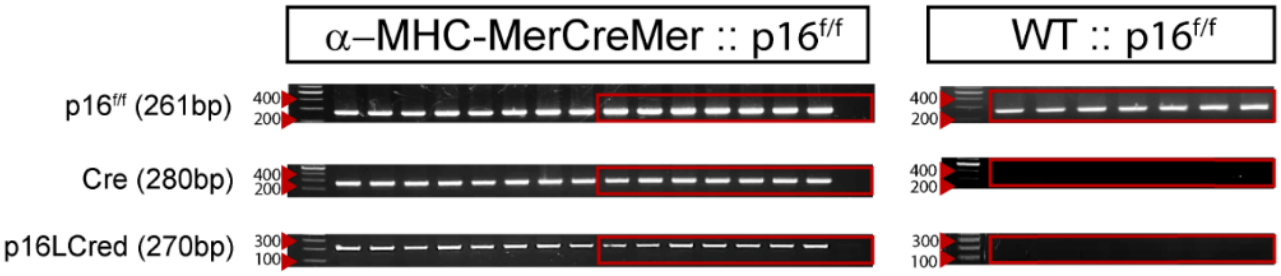

Supplementary Figure 5. **Confirmation of the excision of p16 exon 1α.** Mouse genotypes were established using primers specific to the floxed p16inka4 allele (p16f/f) and αMHC-MercreMer (cre). Bitransgenic MerCreMer-p16f/f mice and WT-p16f/f control mice were given 4-OH-tamoxifen for 14 days. Cre-mediated excision of exon 1α from the MerCreMer :: p16f/f mice was confirmed by PCR using p16LCre primers which span the excised region. Red outline identifies animals used in this study.

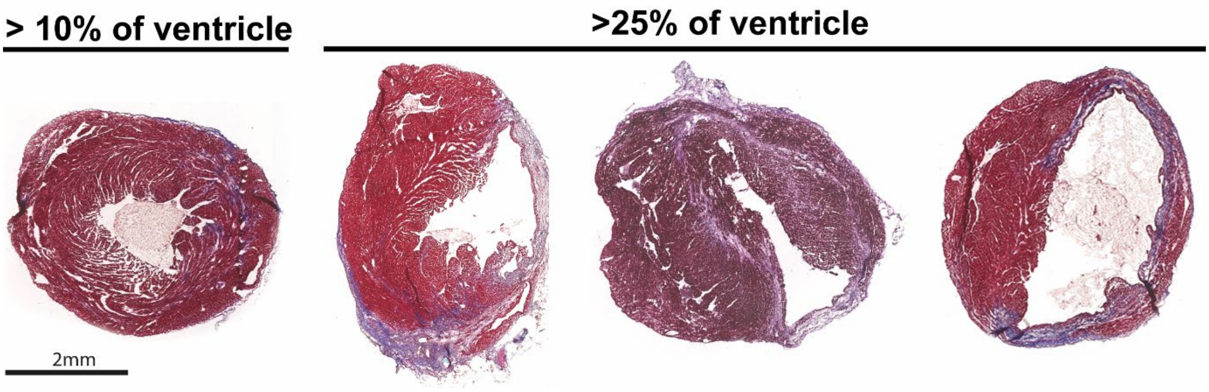

Supplementary Figure 6. **Hearts excluded from study due to incomplete ligation or reperfusion.** Scar size under 10% area of left ventricle indicative of unsuccessful Ligation. Transmural scar which comprises >25% of the left ventricular myocardium indicative of unsuccessful reperfusion.

Cytokine	Concentration (pg/ml ± SD)		
	IR CM-p16^{WT}	IR CM-p16^{KO}	p Value
Eotaxin	0.06 ± 0.10	4.2 ± 7.00	0.18
G-CSF	0.59 ± 0.54	0.07 ± 0.98	0.07
GM-CSF	OOR	OOR	-
IFNγ	14.20 ± 4.2	6.90 ± 6.023	0.08
IL-1α	161.2 ± 54.55	176.9 ± 99.87	0.41
IL-1β	2.78 ± 2.44	2.713 ±3.39	0.49
IL-2	22.30 ± 13.57	20.12 ± 12.08	0.42
IL-3	0.413 ± 0.7	0.05 ± 0.08	0.22
IL-4	0.05 ± 0.03	0.07 ± 0.12	0.41
IL-5	0.026 ± 0.05	OOR	-
IL-6	2.93 ± 0.92	1.477 ± 0.49	0.04 (*)
IL-7	1.75 ± 0.91	1.047 ± 0.90	0.20
IL-9	72.10 ± 19.30	79.36 ± 11.88	0.30
IL-10	31.56 ± 26.88	33.35 ± 20.28	0.47
IL-11	2.21 ± 0.84	1.74 ± 0.46	0.22
IL-12/p40	39.94 ± 27.27	30.91 ± 11.00	0.31
IL-12/p70	1.66 ± 0.675	0.85 ± 1.472	0.22
IL-13	6.287 ± 2.01	5.09 ± 0.94	0.20
IL-15	12.86 ±17.49	24.23 ±12.18	0.36
IL-16	151.9 ± 123.2	138.7 ± 158.9	0.46
IL-17	28.84 ± 17.49	24.23 ± 13.18	0.37
IL-20	OOR	OOR	-
IP-10	3.53 ± 2.36	3.54 ± 1.69	0.50
KC	25.16 ± 15.95	21.32 ± 6.58	0.34
LIF	0.12 ± 0.19	0.15 ± 0.25	0.45
LIX	2753 ± 478.5	2822 ± 1056	0.46
MCP-1	4.107 ± 4.63	2.93 ± 2.80	0.36
MCP-5	5.53 ± 4.252	12.36 ± 8.59	0.12
M-CSF	5.063 ± 2.55	2.55 ± 2.011	0.12
MIG	1.783 ± 0.95	2.50 ± 1.274	0.24
MIP-1a	3.34 ± 3.47	3.1 ± 5.38	0.48
MIP-1β	OOR	OOR	-
MIP-2	29.76 ± 14.09	25.62 ± 19.79	0.39
MIP-3α	3.82 ± 1.34	4.45 ± 0.86	0.26
MIP-3β	0.74 ± 1.29	OOR	-
RANTES	2.183 ± 0.41	1.84 ± 0.86	0.28
TARC	0.46 ± 1.89	1.89 ± 2.26	0.17
TIMP-1	OOR	OOR	-
TNFα	1.35 ± 0.82	0.89 ± 0.96	0.29
VEGF	3.9 ± 3.58	4.9 ± 6.06	0.41
6CKine/Exodus2	306.20 ±194.3	510.86 ± 199.2	0.13
EPO	OOR	OOR	-
Fractalkine	12.82 ± 3.81	12.93 ± 3.09	0.49
MCD	0.77 ± 0.24	0.75 ± 0.074	0.45
IFNB-1	62.12 ± 5.62	60.45 ± 3.81	0.35

Supplementary Table 1. Values from MD-44 Cytokine array. Below detectable range = OOR (Out of Range). Data from n=3 per experimental group. Following tests of normality, data analysed by Student's t-test.
