# Supplementary material for: Senescent cardiomyocytes contribute to cardiac dysfunction following myocardial infarction
Source: NPJ Aging. 2023 Jun 14;9(1):15. doi: 10.1038/s41514-023-00113-5 (PMC10267185; doi:10.1038/s41514-023-00113-5)
Supplement: Supplementary file 2 — Reporting Summary [file 41514_2023_113_MOESM2_ESM.pdf]

nature portfolio

Corresponding author(s): Gavin Richardson

Last updated by author(s): 11/05/2023

Reporting Summary

Nature Portfolio wishes to improve the reproducibility of the work that we publish. This form provides structure for consistency and transparency in reporting. For further information on Nature Portfolio policies, see our Editorial Policies and the Editorial Policy Checklist.

Statistics

For all statistical analyses, confirm that the following items are present in the figure legend, table legend, main text, or Methods section.

n/a	Confirmed	
☐	☒	The exact sample size (*n*) for each experimental group/condition, given as a discrete number and unit of measurement
☐	☒	A statement on whether measurements were taken from distinct samples or whether the same sample was measured repeatedly
☐	☒	The statistical test(s) used AND whether they are one- or two-sided<br>*Only common tests should be described solely by name; describe more complex techniques in the Methods section.*
☐	☒	A description of all covariates tested
☐	☒	A description of any assumptions or corrections, such as tests of normality and adjustment for multiple comparisons
☐	☒	A full description of the statistical parameters including central tendency (e.g. means) or other basic estimates (e.g. regression coefficient) AND variation (e.g. standard deviation) or associated estimates of uncertainty (e.g. confidence intervals)
☐	☒	For null hypothesis testing, the test statistic (e.g. F, t, r) with confidence intervals, effect sizes, degrees of freedom and P value noted<br>*Give P values as exact values whenever suitable.*
☐	☒	For Bayesian analysis, information on the choice of priors and Markov chain Monte Carlo settings
☐	☒	For hierarchical and complex designs, identification of the appropriate level for tests and full reporting of outcomes
☐	☒	Estimates of effect sizes (e.g. Cohen's *d*, Pearson's *r*), indicating how they were calculated

Our web collection on statistics for biologists contains articles on many of the points above.

Software and code

Policy information about availability of computer code

Data collection	All images were acquired using Axio Imager (Zeiss) and analysed using ZEN 2.3 (Zeiss).<br>Magnetic resonance images were acquired using a horizontal bore 7.0T Varian microimaging system (Varian Inc., Palo Alto, CA, USA)
Data analysis	All quantifications were performed blinded to treatment and genotype using digital image analysis (ImageJ; U.S. National Institutes of Health; http://rsbweb.nih.gov/ij/).<br>Statistical analysis was performed using Graphpad prism (Dotmatrics)

For manuscripts utilizing custom algorithms or software that are central to the research but not yet described in published literature, software must be made available to editors and reviewers. We strongly encourage code deposition in a community repository (e.g. GitHub). See the Nature Portfolio guidelines for submitting code & software for further information.

Data

Policy information about availability of data

All manuscripts must include a data availability statement. This statement should provide the following information, where applicable:

- Accession codes, unique identifiers, or web links for publicly available datasets
- A description of any restrictions on data availability
- For clinical datasets or third party data, please ensure that the statement adheres to our policy

All data generated or analysed during this study are included in this published article and its supplementary information files.

Research involving human participants, their data, or biological material

Policy information about studies with human participants or human data. See also policy information about sex, gender (identity/presentation), and sexual orientation and race, ethnicity and racism.

Reporting on sex and gender	NA
Reporting on race, ethnicity, or other socially relevant groupings	NA
Population characteristics	NA
Recruitment	NA
Ethics oversight	NA

Note that full information on the approval of the study protocol must also be provided in the manuscript.

Field-specific reporting

Please select the one below that is the best fit for your research. If you are not sure, read the appropriate sections before making your selection.

☒ Life sciences ☐ Behavioural & social sciences ☐ Ecological, evolutionary & environmental sciences

For a reference copy of the document with all sections, see nature.com/documents/nr-reporting-summary-flat.pdf

Life sciences study design

All studies must disclose on these points even when the disclosure is negative.

Sample size	Sample size estimates were originally calculated a sample size for power value of 80% and a confidence of 95% we have ascertained that the studies requiring the largest sample size are the MRI measurements of cardiac function.
Data exclusions	As described in the manuscript, to control for a lack of total occlusion or incomplete reperfusion following 60 min ligation, prior to any other analysis, scar size was measured in all animals in a blinded fashion and animals with a scar size of >25% or <10% were excluded from all analysis all heart removed from the study are shown in Supplementary Figure 5.
Replication	All experiments were performed using an individual mouse for each biological replicate. Additional, as discribed in the methods mutiple images and measurements were obtained from each biological repeat.
Randomization	Mice were genotyped to obtain sufficient animal numbers (based on power calculations). The genotype was then blinded and animlas were randomized groups for LAD-ligation surgery. Mice genotype remained blinded until all analysis was complete.
Blinding	Investigators were blinded for all analysis.

Reporting for specific materials, systems and methods

We require information from authors about some types of materials, experimental systems and methods used in many studies. Here, indicate whether each material, system or method listed is relevant to your study. If you are not sure if a list item applies to your research, read the appropriate section before selecting a response.

Materials & experimental systems

n/a	Involved in the study	
☐	☒	Antibodies
☒	☐	Eukaryotic cell lines
☒	☐	Palaeontology and archaeology
☐	☒	Animals and other organisms
☒	☐	Clinical data
☒	☐	Dual use research of concern
☒	☐	Plants

Methods

n/a	Involved in the study	
☒	☐	ChIP-seq
☒	☐	Flow cytometry
☒	☐	MRI-based neuroimaging

Antibodies

Antibodies used	1) rat ant-p21 (Hugo291, Abcam),<br>2)anti-Troponin C (ab137130, Abcam),<br>3)rabbit anti-p16Ink4a (100401170, Rockland)
Validation	1) Product website: https://www.abcam.com/products/primary-antibodies/p21-antibody-hugo291-ab107099.html?productWallTab=ShowAll. Referenced 24 times<br>2) Product Website: https://www.abcam.com/products/primary-antibodies/tnnc1-antibody-epr9692b-ab137130.html. Referenced 3 times.<br>Abcam standards webpage. https://www.abcam.com/primary-antibodies/recombinant-antibodies<br><br>3) https://www.rockland.com/categories/primary-antibodies/p16-antibody-100-401-170/. 2 references. We have prevoulsy demonstrated the specificty of this antibody using p16 knock-out mice. https://doi.org/10.1111/acel.13249

Animals and other research organisms

Policy information about studies involving animals; ARRIVE guidelines recommended for reporting animal research, and Sex and Gender in Research

Laboratory animals	Cdkn2atm2.1Nesh line mice were crossed with Myh6-cre/Esr1.
Wild animals	No Wild animals were used
Reporting on sex	As a sex difference in the response to MI has been observed in mice only male mice were used in this study. https://doi.org/10.3390/ijms24076443
Field-collected samples	None
Ethics oversight	Ethics approval statement: All animal studies were conducted in accordance with the Guidance on the Operation of the Animals (Scientific Procedures) Act, 1986 (UK Home Office), and approved by the local ethics committee (Animal Welfare & Ethical Review Body) at Newcastle University.

Note that full information on the approval of the study protocol must also be provided in the manuscript.
